# Comparative study on guidelines in determining HBV phases in Iranian patients 

**Published:** 2019

**Authors:** Sara Ashtari, Afsaneh Sharifian, Behzad Hatami, Seyed Reza Mohebbi, Gholamreza Nouri, Monireh Bazdar, Nosratollah Naderi

**Affiliations:** 1 *Basic and Molecular Epidemiology of Gastrointestinal Disorders Research Center, Research Institute for Gastroenterology and Liver Diseases, Shahid Beheshti University of Medical Sciences, Tehran, Iran*; 2 *Gastroenterology and Liver Diseases Research Center, Research Institute for Gastroenterology and Liver Diseases, Shahid Beheshti University of Medical Sciences, Tehran, Iran*; 3 *Foodborne and Waterborne Diseases Research Center, Research Institute for Gastroenterology and Liver diseases, Shahid Beheshti University of Medical Sciences, Tehran, Iran*

**Keywords:** Chronic hepatitis B, ALT level, CHB phases, Iran

## Abstract

**Aim:**

The aim of this study was to compare the different phases of chronic Hepatitis B virus (HBV) infection with different values for normal ALT.

**Background:**

For many years, the upper limit of 40 IU was considered normal for ALT for both sexes, but in recent years this value is challenged and some guidelines have lowered their limit.

**Methods:**

In this cross-sectional study, 2000 HBsAg positive patients who were referred to Taleghani Hospital, Tehran, Iran, from 2011 through 2018 were classified in four groups according to American Association of the study of the liver disease (AASLD), European Association of the study of the liver (EASL) /Asian-Pacific Association of the study of the liver (APASL) and American Collage of Gastroenterology (ACG) guidelines. The frequency of each group based on 3 different guidelines was compared.

**Results:**

In HBeAg positive patients (n=100), the percentage of immune tolerance phase was 43% according to AASLD cutoff for normal ALT (35 IU for men, 25 IU for women), while it was 68% and 28% with regard to EASL/APASL and ACG (30 IU for men, 19 IU for women) cutoffs respectively. In HBeAg negative patients (n=1900), 66.68% were inactive carriers according to AASLD, but the percentage changed to 82.89% and 52.42% considering EASL/APASL and ACG values, respectively.

**Conclusion:**

Using ACG and to a lesser extent AASLD cutoff for ALT, many patients shift from immune tolerance and inactive carrier state into the immune active phase. Thus, more patients are candidates for treatment or intensive workup to determine the extent of liver damage.

## Introduction

 Hepatitis B virus (HBV) infection still remains as a major global health problem. In spite of available vaccination, there is still no cure for chronic HBV (CHB) infection and we can only manage it. CHB can cause chronic hepatitis, cirrhosis, and hepatocellular carcinoma (HCC) (1). The global prevalence of HBV varies geographically; from< 2% in Western Europe, North America and Australia to > 8% in Africa and Asia. Iran is a Middle Eastern country, but due to mass vaccination since 1933, the prevalence of HBV infection has declined from 2%-4% to 1.7% (1.3%-0.8% among children and adolescents) (2-5). Nevertheless, we have to keep in mind that the prevalence of HBV is still within 2-4% in middle and old aged Iranians; i.e. nearly two million Iranian adults are chronically infected with HBV (6, 7). 

The natural history of CHB is variable and affected by complex interaction between host, viral, and environmental factors (8). Taking into account HBV DNA levels (viral loads), alanine aminotransferase (ALT) levels, hepatitis B e antigen (HBeAg) status and finally the presence or absence of liver inflammation, chronic HBV infection can be classified into different clinical phases (9, 10). These phases outline our approach to appropriate management of these patients. There are 3 major international guidelines for HBV over the world; American Association of the study of the liver disease (AASLD) (11), European Association of the study of the liver (EASL) (12), and Asian-Pacific Association of the study of the liver (APASL) (13). Normal ALT level is 40 (IU/ml) in EASL and APASL for both sexes, while it is 35 IU for men and 25 IU for women in AALSD. EASL has also new terms for CHB phases which lowers the threshold for treatment. According to ACG (American Collage of Gastroenterology) guideline for evaluation of abnormal liver chemistries, true healthy normal ALT level ranges from 29 to 33 IU/l for males and 19 to 25 IU/l for females (14). Table 1 describes definitions for phases of CHB according to different guidelines.

The aim of this study is to compare these phases with different cutoffs for normal ALT and discover if these differences can lead to considerable changes in our care for HBV-infected patients. 

## Methods

**Patient population**

In this cross-sectional study, 2000 CHB patients who were referred to Taleghani Hospital (one of the Iran’s main referral centers for liver diseases) from different parts of Iran through 2011 till 2018 were enrolled. All of them had positive HBsAg for at least 6 months and gave their written informed consent to participate in this study. The patient’s demographic information including name, age, sex and laboratory tests were registered in data collection forms by a trained researcher. This study was approved by the Ethics Committee of Research Institute for Gastroenterology and liver diseases, Shahid Beheshti University of Medical Sciences. 

**Laboratory methods**

Four milliliters of patient’s blood samples were added to EDTA tube for serum separation and tested by ELISA method for ALT levels. Phenol-chloroform extraction method was used to extract DNA from blood samples. The extracted DNA was stored at -20°C, before performing the HBV DNA quantitative PCR. HBV cDNA was amplified by PCR and HBV DNA was amplified by semi-nested PCR and then the fragments were detected on 1% w/v agarose gel. The gels were examined by UV transillumination or white light. If samples had detectable HBV DNA, quantitative real-time PCR was done to determine the level of DNA.

**Statistical methods**

Qualitative variables were reported as the number or percentage of different clinical phases of CHB based on three ALT values. All analyses were performed using SPSS version 21.0 (SPSS INC, Chicago, IL, USA). 

## Results

From 2000 patients in our study, 100 were HBeAg positive and 1900 were HBeAg negative. In HBeAg positive patients, the percentage of immune tolerance phase was 43% according to AASLD cutoff for ALT, while it was 68% and 28% with regard to EASL/APASL and ACG values respectively. In HBeAg negative patients, 66.68% were inactive carriers according to AASLD values, but this percentage was 82.89% and 52.42% considering EASL/APASL and ACG values respectively (Table 2).

**Table 1:** Comparison of CHB phases in 3 guidelines (AASLD, EASL and APSLD)

HBeAg	+	+	-	-
HBeAb	-	-	+	+
ALT	N	× 1-2	N	× 1-2
HBV DNA (IU/ml)	Very High	High( >20000)	Low (< 2000)	Fluctuating>2000
AASLD 2018	Immune tolerance	Immune active	Inactive HBsAg carrier	Immune active
EASL 2017	HBeAg positive CHB infection	HBeAg positive CHB hepatitis	HBeAg negative CHB infection	HBeAg negative CHB hepatitis
APASLD 2016	Immune tolerance	Immune active	Low replicative	Reactivation

**Table 2:** Comparison of percentage of CHB phases due to different guidelines

Phases Guidelines	^1,3^Immune tolerance ^2^ HBeAg positive CHB infection ,3licativeion APSLD)ines or fibrosise added to EDTA tube for serum sepretion.es leads to cosiderable	^1,3^Immune active ^2^ HBeAg positive CHB hepatitis	^1^Inactive carrier ^2^HBeAg negative CHB infection ^3^Low replicative	^1^Immune active ^2^HBeAg negative CHB hepatitis ^3^Reactivation
AASLD^1^	43 (2.15%)	57(2.85%)	1267(63.35%)	633 (31.65%)
EASL^2^/APASL^3^	68 (3.4%)	32 (1.6%)	1575 (78.75%)	325 (16.25%)
ACG^4^	28 (1.4%)	72 (3.6%)	996 (49.8%)	904 (45.2%)

^1^According to AASLD 2018, ^2^According to EASL 2017, ^3^According to APASL 2015

## Discussion

CHB infection is a disease with substantial economic burden for payers, patients, and community. CHB, especially in extreme cases, can be very expensive, while appropriate management of these patients at early stages may prevent progression of disease and improve their quality of life and reduce expenses. In Iran in 2015, it was estimated that the total annual costs for the population of active CHB patients and for those receiving treatment at various disease stages were 450 million and 226 million dollars, with 64% and 70% allocated to direct while 36% and 30% to indirect costs respectively (15). Therefore, HBV infection has still a significant economic burden on the health system where its natural course and different phases play a significant role in estimating these needs (financial, laboratory equipment, human resources) for policy making and better management of this disease. Patients with normal ALT levels usually do not need treatment, and follow-up with periodic reevaluations is enough. On the other hand, patients with ALT levels 1-2 times the normal limit generally are considered as grey zone and need more dedicated workups and even liver biopsy, while those with ALT levels more than 2 times the normal range in general benefit from treatment (11, 13, 16). In this study, the majority of our patients were HBeAg negative (95%) which is significantly more than those reported in previous years in Iran (17-19). Meanwhile, it is completely consistent with recent studies in the world such as; Europe, Asia, and the United States reporting an ascending trend toward HBeAg negative CHB patients’ prevalence over HBeAg positive patients (20-24). This shift may be related to several factors including the successful vaccination programs, improved screening methods and effective treatment strategies and mutations in pre C region of the virus (25-27). In our study, according to AASLD and EASL/APSL guidelines, more than two thirds of patients were inactive carriers who do not need treatment. However, when the cutoff of ALT falls to ACG values, this percentage drops to near 50% and instead the percentage of immune active patients who do need treatment rises to near 50%. These differences still exist (though with less significance) when we compare AASLD to EASL/APASL values. This is a very important issue, as it shows that many patients that have been labeled as inactive carrier may indeed have active disease and benefit from treatment. It is well known that treatment can prevent or slow down progression of liver injury and is one of the main strategies to prevent HCC. The incidence of HCC is less than 2% in Iran (28), but it is estimated that HBV is responsible for 50% to 80% of HCC cases (29-31). Thus, treating these patients can help reduce the rates of cirrhosis and HCC in future. On the other hand, these changes lead to increased immediate costs of HBV management. As such, it seems necessary to asses if these rises in expenses are justified and can lead to diminished HBV’s economic burden in the long run.
